# Cholera outbreak in southern Tanzania: risk factors and patterns of transmission.

**DOI:** 10.3201/eid0707.010741

**Published:** 2001

**Authors:** C J Acosta, C M Galindo, J Kimario, K Senkoro, H Urassa, C Casals, M Corachán, N Eseko, M Tanner, H Mshinda, F Lwilla, J Vila, P L Alonso

**Affiliations:** Ifakara Health Research and Development Centre, Ifakara, Tanzania.

## Abstract

To identify risk factors and describe the pattern of spread of the 1997 cholera epidemic in a rural area (Ifakara) in southern Tanzania, we conducted a prospective hospital-based, matched case- control study, with analysis based on the first 180 cases and 360 matched controls. Bathing in the river, long distance to water source, and eating dried fish were significantly associated with risk for cholera. Toxigenic Vibrio cholerae O1, biotype El Tor, serotype Ogawa, was isolated in samples from Ifakara's main water source and patients' stools. DNA molecular analyses showed identical patterns for all isolates.


### Cholera Outbreak in Southern Tanzania: Risk Factors and Patterns of Transmission

**Camilo J. Acosta,*† Claudia M. Galindo,*† John Kimario,* Kesheni Senkoro,* Honorathy Urassa,* Climent Casals,† Manuel Corachán,† N. Eseko,‡ Marcel Tanner,§ Hassan Mshinda,* Fred Lwilla,¶ Jordi Vila,† and Pedro L. Alonso†**

*Ifakara Health Research and Development Centre, Ifakara, Tanzania; †Hospital Clínic, Barcelona, Spain; ‡Ministry of Health, Dar es Salaam, Tanzania; §Swiss Tropical Institute, Basel, Switzerland; and ¶District Medical Office, Kilombero, Morogoro Region, Tanzania

To identify risk factors and describe the pattern of spread of the 1997 cholera epidemic in a rural area (Ifakara) in southern Tanzania, we conducted a prospective hospital-based, matched case-control study, with analysis based on the first 180 cases and 360 matched controls. Bathing in the river, long distance to water source, and eating dried fish were significantly associated with risk for cholera. Toxigenic *Vibrio cholerae* O1, biotype El Tor, serotype Ogawa, was isolated in samples from Ifakara's main water source and patients' stools. DNA molecular analyses showed identical patterns for all isolates.

The reemergence of cholera is presenting unprecedented challenges (1,2). Since the seventh pandemic caused by *Vibrio cholerae* biotype El Tor began in Indonesia in 1961, most regions of the world continue to report cholera. 1997 was marked by a cholera epidemic affecting most countries in East Africa, with spread toward central and southern parts of the continent. Africa reported 118,349 cases to the World Health Organization in 1997, for 80% of cases worldwide. Africa also had the highest overall case-fatality rate (4.9%), compared with 1.3% in the Americas and 1.7% in Asia (3). Tanzania has consistently reported cholera cases; annual reports ranged from 1,671 cases in 1977 to 18,526 in 1992 (4,5). During the last 2 decades, three major cholera epidemics have occurred: 1977-78, 1992, and 1997 (3-5). In 1997, Tanzania had one of the highest case-fatality rates in East Africa (5.6%), with 2,268 deaths in 40,226 cases (3). We describe risk factors and pattern of spread of the 1997 cholera epidemic in a rural area in southern Tanzania.

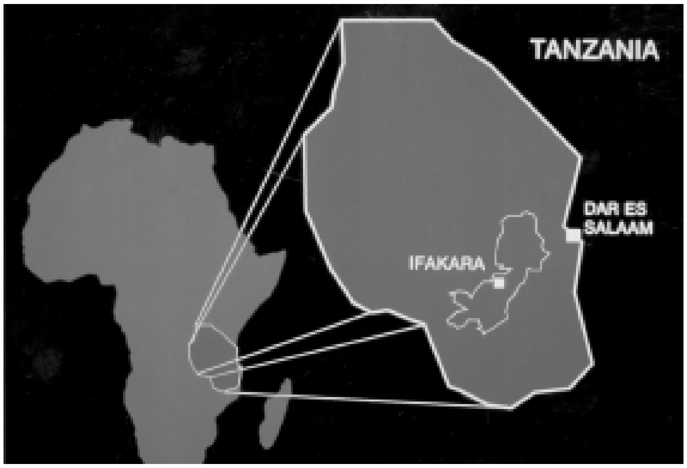

Figure 1. Map of Tanzania showing Ifakara.

### Methods

Ifakara is 270 m above sea level in the Kilombero District river valley in southeastern Tanzania (08ºS; 32ºE) (Figure 1). It is a rural area (estimated population 57,000), and most inhabitants are subsistence farmers growing rice and maize. Fishing is also common. The town is crossed by the Lumemo River, a tributary of the Kilombero River. There are two rainy seasons: March through May, and December through January (1997 rainfall 1,439 mm). A short, cool dry season follows the long rains in June and July.

In Ifakara, the last cholera epidemic was in 1992. Most outpatient and admissions occur at Ifakara's Saint Francis Designated District Hospital (SFDDH), a 375-bed, district referral hospital. Demand-driven research takes place in an adequately equipped, district-based international research center, the Ifakara Health Research and Development Centre (IHRDC).

### The Outbreak

An increase in acute severe diarrhea cases in adults was noted in early June 1997 at SFDDH. On June 23, the first cholera case was confirmed by culture. At that time, a cholera control campaign was launched, following standard recommendations (6,7). As part of control activities, an outbreak investigation was begun, and two cholera treatment sites were established. One was near the area where the outbreak was first noticed, the Lumemo River; the other site was in a ward at the district hospital.

The outbreak investigation consisted of a prospective hospital-based, case-control study designed to identify cholera risk factors. A case was defined as illness in a patient

Address for correspondence: Pedro L. Alonso, Hospital Clínic, Villarroel 170, E-08036 Barcelona, Spain; fax: 3493-451-5272; e-mail: alonso@medicina.ub.es

>5 years of age, visiting either of the cholera sites from June 23 to December 31, 1997, and hospitalized with acute onset of watery diarrhea. For each case, two age- and sex-matched controls were selected and interviewed on the same day as the case interview. Controls were selected from patients admitted to the district hospital within 2 days of the date of case admission. Data on possible risk factors were documented by a standardized questionnaire (Table 1). After preliminary analysis of the first 60 cases and 120 controls, access to the Lumemo River was restricted. The final analysis was based on 180 cholera cases and 360 controls. We evaluated possible sources of bias 1 year later by interviewing a random sample of 100 community controls matched by age, sex, and neighborhood. This sampling procedure was a two-stage cluster (10 families per cell unit) intended to assess prevalence of cholera risk factors at the community level.

The distribution of cholera cases in relation to the main water source was determined by mapping the distance from ill persons' houses to the Lumemo River. Mapping was facilitated by use of a GeoExplorer II Mapping system and two Trimble Global Positioning System receivers (Trimble, Liverpool, United Kingdom). Differentially corrected positions were then processed with PFINDER software. To assess whether cases were equally distributed outwards from the river, areas were mapped at 250-m intervals. Four major zones were used, two on each side of the river; these zones were used for sampling rather than streets, which are not well defined in Ifakara. Zone-specific attack rates were calculated as cases per 1,000 residents for the duration of the outbreak, after a house-to-house census of all residents living within 500 m of the river was conducted.

Fecal specimens and rectal swabs were obtained from all patients admitted to the treatment sites until 60 positive cultures were obtained. Samples were obtained at the beginning of the epidemic and 1 month later from two water sources (the Lumemo River and the water pump closest to the river) and were sent to the IHRDC laboratory. Each specimen was immediately processed and plated onto thiosulfate-citrate-bile salt-sucrose agar. Sucrose-positive colonies were further tested on the basis of standard biochemical reactions (8). *V. cholerae* isolates were agglutinated with polyvalent 01 and monospecific Ogawa, Inaba, and Hikojima antisera (Difco Laboratories, Madrid, Spain). The isolates were tested for susceptibility to seven antibacterial drugs by disk diffusion (9). Susceptibility testing was done for the following antibiotics: ampicillin, 30 mg per disk; chloramphenicol, 60 mg; ciprofloxacin, 10 mg; gentamicin, 40 mg; nalidixic acid, 130 mg; tetracycline, 10 mg; and trimethoprim-sulfamethoxazole, 5.2 mg and 240 mg, respectively.

Polymerase chain reaction (PCR) was used to detect the *ctx* gene for all isolates. The primers used were specific for the *V. cholerae* enterotoxin (Takara Shuzo Co. Ltd., Otsu, Shiga, Japan). The analysis of chromosomal DNA by digestion with low-frequency-of-cleavage restriction enzymes and separation by pulsed-field gel electrophoresis (PFGE) was done with the *Not* I enzyme on a CHEF-DR IIII system (Bio-Rad Laboratories, Richmond, CA).

Data were double-entered into FoxPro databases (Microsoft Corp.) and checked for range, internal consistency, and referential integrity. Statistical analysis was performed with STATA statistical software (Stata Corp., 1997). McNemar's chi-square test was used for the univariate analysis. Variables significantly associated with cholera at or below the 5% level in the univariate analysis were included in the modeling. Multivariate conditional logistic regression was performed, the final model was fitted, and attributable risk percentages were calculated (Table 2).

### Results

From June 23 through December 31, a total of 785 patients were admitted to the Ifakara cholera treatment sites. The number of cases peaked between June 30 and July 4 (Figure 2); 369 (47%) were males; 376 (48%) of the cholera patients

Table 1. Univariate analysis results of potential risk factors during the 5 days before onset of illness in cases and matched controls

| Risk factors | Cases, % (n=180) | Controls exposed % (n=360) | Matched odds ratio | 95% CI | p-value |
|---|---|---|---|---|---|
| Social activities | | | | | |
| Attended funeral recently | 6.2 | 2.2 | 2.7 | 1.1-6.8 | 0.03 |
| Attended party recently | 2.8 | 4.7 | 0.6 | 0.2-1.7 | 0.33 |
| Travel in the past 2 weeks | 19.0 | 13.6 | 1.5 | 0.9-2.5 | 0.10 |
| Standard of living | | | | | |
| Mud housing[a] | 48.0 | 36.0 | 1.7 | 1.2-2.5 | 0.01 |
| Non-iron sheet roof | 55.6 | 39.2 | 2.0 | 1.4-2.8 | 0.00 |
| No latrine at home | 56.6 | 9.8 | 11.4 | 6.3-20.5 | 0.00 |
| Simple pit latrine[b] | 91.4 | 81.2 | 2.7 | 1.4-5.1 | 0.001 |
| Water exposure | | | | | |
| >10 minutes to water source | 26.1 | 11.4 | 2.8 | 1.7-4.6 | 0.00 |
| Unboiled drinking water | 86.7 | 79.2 | 1.9 | 1.1-3.2 | 0.02 |
| Unfiltered drinking water | 89.4 | 84.2 | 1.7 | 0.9-3.0 | 0.07 |
| River bathing | 56.6 | 9.8 | 11.4 | 6.3-20.5 | 0.00 |
| Inside tap water[c] | 18.9 | 9.4 | 3.0 | 1.7-5.1 | 0.00 |
| Other source water[c] | 18.3 | 7.5 | 3.7 | 2.0-6.7 | 0.00 |
| Food Exposures | | | | | |
| Dried fish | 52.5 | 7.2 | 13.0 | 7.3-23.3 | 0.00 |
| Prawns | 13.9 | 5.0 | 3.1 | 1.6-5.9 | 0.00 |
| Uncooked vegetables | 25.1 | 20.1 | 1.3 | 0.9-2.0 | 0.20 |
| Fruits | 32.4 | 30.4 | 1.1 | 0.7-1.6 | 0.60 |
| Religion[d] | 41.2 | 23.1 | 2.3 | 1.1-3.4 | 0.00 |

[a]Compared with brick houses.
[b]Compared with ventilated improved pit latrines.
[c]Compared with hand-pumped drinking water.
[d]Muslims (compared with other religions, including Catholics, Protestants, and others).

Table 2. Multivariate analysis of risk factors for cholera in 1997 epidemic, Ifakara, Tanzania

| Risk factor | Odds ratio | p-value | 95% CI[a] | Attributable risk | (%) |
|---|---|---|---|---|---|
| >10 minutes to water source | 2.7 | 0.00 | 1.7-4.4 | 63[b] | 17[c] |
| River bathing | 14.4 | 0.00 | 8.8-23.5 | 93[b] | 49[c] |
| Eating dried fish | 12.1 | 0.00 | 7.7-19.1 | 92[b] | 52[c] |

[a]CI = confidence interval.
[b]The attributable fraction among the exposed population, an estimate of the proportion of exposed cases attributable to exposure.
[c]The population-attributable fraction, which is the net proportion of all cases attributable to exposure.

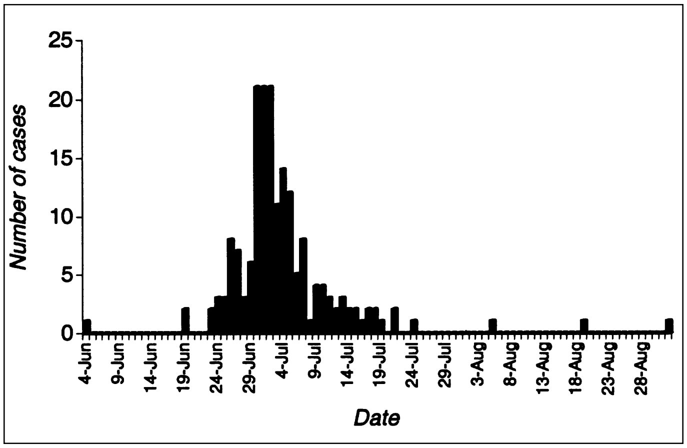

Figure 2. Distribution of disease onset by dates, in patients admitted for acute watery diarrhea to cholera treatment sites in Ifakara in 1997.

Table 3. Prevalence of cholera risk factors in Ifakara, Tanzania

| Risk factors (exposures) | Cholera cases n=180 | Hospital controls n=360 | Community controls n=100 |
|---|---|---|---|
| Activities | | | |
| Attended funeral recently | 6.2 | 2.2 | 60.6 |
| Attended party recently | 2.8 | 4.7 | 88.5 |
| Travel in the past 2 weeks | 19.0 | 13.6 | 14.4 |
| Fishing | 54.0 | 51.0 | 53.8 |
| Water | | | |
| >10 minutes to water source | 26.1 | 11.4 | 14.4 |
| Unboiled drinking water | 86.7 | 79.2 | 90.4 |
| Unfiltered drinking water | 89.4 | 84.2 | 92.3 |
| River bathing | 56.6 | 9.8 | 33.7 |
| Food | | | |
| Dried fish | 52.5 | 7.2 | 76.9 |
| Prawns | 13.9 | 5.0 | 14.4 |
| Uncooked vegetables | 25.1 | 20.1 | 29.8 |
| Fruits | 32.4 | 30.4 | 73.1 |

were <25 years of age. Seventeen deaths were reported (case-fatality rate 2.1%). Zone-specific attack rates were from 11 (west zone) to 41 cases per 1,000 residents (east zone), showing an increase in the densely populated zones of the town.

In univariate analysis of recent exposure to potential risk factors, low standards of living (indicated by a mud house, having a thatched roof, and having simple pit latrine or none) affected significantly the likelihood of disease (Table 1). Risk factors for exposure to water, including long distance to water source (>10 minutes walk), use of unboiled water, bathing in the river, and the use of tap water inside the house instead of pumped water, were all significantly associated with cholera, as well as having recently eaten dried fish and prawns. Multivariate analyses showed that distance to the water source (odds ratio [OR] 2.7; 95% confidence interval [CI] 1.7-4.4), having eaten dried fish recently (OR = 12.1; 95% CI 7.7-19.1), and bathing in the river (OR = 14.4; 95% CI 8.8-23.5) were independently significantly associated with risk for cholera (Table 2). Interaction terms neither changed nor improved the fit of the model.

The attributable risk percentages (Table 2) provide an approximate summary of the importance of each risk factor, taking into consideration both the strength of the association and its prevalence; these estimates assume that all confounding variables were measured and no sources of bias exist. For example, river bathing accounted for 93% of all cases among the population with this exposure, and 49% of all cases could have been averted by preventing this practice.

Cholera patients were more likely to be exposed to bathing in the river than community controls ($p<0.01$) but hospital controls were less likely to be exposed than 100 community controls ($p<0.01$) (Table 3). Cholera cases were more likely to walk >10 minutes to water sources than community controls ($p<0.05$).

*V. cholerae* was isolated from stool specimens and water samples from the Lumemo River, but not from any other water source. All isolates were toxigenic *V. cholerae* O1, biotype El Tor, serotype Ogawa; DNA genotyped by PCR-PFGE in samples isolated from the river and patient stools had identical patterns (Figure 3). All isolates were susceptible to tetracycline, ampicillin, amoxycillin + clavulanic acid, nitrofurantoines, and quinolones and were resistant to cotrimoxazole.

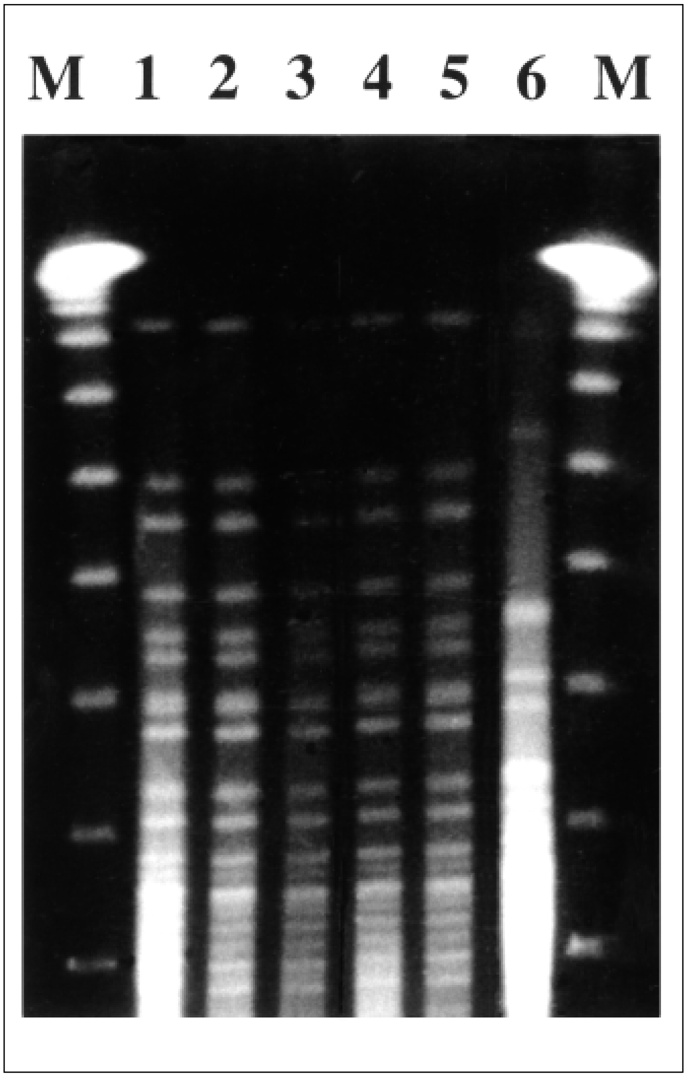

Figure 3. Pulsed-field gel electrophoresis patterns of *Vibrio cholerae* isolates. DNA molecular weight markers (M lanes); *V. cholerae* strain isolated from water (Lane 1); *V. cholerae* showing isolates from patients (Lanes 2-5); *V. cholerae* strain 01 El Tor from the Spanish type culture collection (Lane 6).

### Conclusion

This hospital-based, case-control study identified three risk factors independently associated with the spread of the 1997 cholera outbreak in Ifakara, rural Tanzania. These factors were bathing in the river, eating dried fish, and living >10 minutes walking distance from the closest water source. Data from zone-specific attack rates also suggest an increase in cholera cases in the densely populated zones of the town.

Bathing in Ifakara's principal water source, the Lumemo River, had an independent, statistically significant OR of 14.4. More than 90% of river bathers were ill, and approximately 50% of these cases could have been prevented if access to the river had been closed. Moreover, identical strains, based on the DNA typing, were isolated both from the patient stools and river water, as previously reported in Bangladesh and the 1992 epidemic in Burundi (10,11). After the 1997 outbreak in Ifakara, the largest town in Kilombero District, most areas of the district were affected by the epidemic. Cases in other areas in the district began to be reported on September 2, reaching 134 by December 1997 and >1,000 by September 1998 (Ministry of Health, unpub. data). In the town of Itete, we were able to follow the beginning of the outbreak, which appeared to have been due to contamination of the Itete River with clothes of a cholera adult patient who came from Ifakara town and died in Itete. The epidemic in Ifakara was preceded by a funeral near the Lumemo River, which travelers from Dar es Salaam had attended (data not shown). Indeed, the last three cholera epidemics in Ifakara have been preceded by epidemics in Dar es Salaam (5).

Long distance to water source, defined as >10 minutes walk, was also independently associated with risk for cholera. Twenty-six percent of cases and 11% of controls had to walk >10 minutes to collect water. Long distance may be an indirect measure of poor hygienic habits caused by scarcity of water. In addition, this link may be indirectly associated with drinking water from the river: 23% of cholera patients living >10 minutes walking distance from a water source and 9% of controls actually collected water from the river. The other 77% and 91%, respectively, collected water from a pump or tap. Persons living farther away from the river may store water for longer periods, thus facilitating *V. cholerae* growth; risk for cholera is dependent on inoculum size (12,13).

Eating dried fish in Ifakara also seemed to be a cholera risk factor. Although to our knowledge, this is the first time a foodborne exposure has been identified as a cholera risk factor in East Africa, this association should be interpreted with caution. We were unable to isolate *V. cholerae* in dried fish samples, and hospital controls ate less dry fish than community controls (Table 3). However, hospital controls may have been more likely to recall food served at the hospital, where fish is rarely offered.

These associations could also be explained by different sources of bias. At the design stage, we asked questions to identify possible sources of bias. First, we asked questions about activities such as recent travel or attendance at a party. We aimed to identify whether hospital controls, as a result of their cause of admission, were as capable of bathing in the river as patients. We then assumed that this increased risk could not have resulted from selecting hospital controls who were physically incapable of bathing in the river, since they were equally capable of having attended a party or traveled recently. Additionally, we recruited only recently admitted patients (within 2 days of admission). Second, we included prawns in the questionnaire. Since prawns are never offered at the hospital, if food availability at the hospital were the underlying reason for the fish-cholera association, prawns should be associated as well. However, multivariate analysis showed only fish to be associated. The extent to which this fish-cholera association may be due to bias remains unclear.

The hospital-based case-control study, which offers the advantage of lower cost compared with a community-based design, seemed to be a reasonable approach for identifying risk factors such as bathing in the river and distance to water source. However, it may lead to over-estimation of the strength of the association, and its role in identifying food as risk factor is debatable.

In conclusion, in Ifakara, as reported previously in Tanzania (14), cholera transmission seems to have been predominantly waterborne. The 1997 Ifakara cholera outbreak could have been initiated by river contamination and facilitated by poor sanitation and lack of safe water. Control measures such as blocking access to the river and surveillance of travelers to avoid contamination of water sources seem to be impractical. Besides other control measures described elsewhere for African settings (15), trying to control cholera in densely populated cities such as Dar es Salaam may help control its spread. In the absence of adequate sanitation and water supply in less developed countries, health education campaigns stressing the need for safe water, food handling, and prompt medical care remain feasible options to reduce disease and death from cholera in rural East Africa.

### Acknowledgments

We thank the staff of the Ifakara Health Research and Development Centre, with special consideration for the hard work of the clinical officers and the clinical and laboratory technicians of Saint Francis Designated District Hospital; M. Mbonja for collecting and providing official cholera records; and P. Kibatala and H. Masanja for their support.

This study was funded by the Spanish Agency for International Cooperation.

Dr. Acosta is a research scientist for the International Vaccine Institute in Seoul, South Korea. He has coordinated malaria and tuberculosis intervention trials for the Ifakara Health Research and Development Centre in Tanzania, which is affiliated with the Unidad de Epidemiología, Hospital Clinic of Barcelona.

### References

1. Frenk J, Sepulveda J, Gomez-Dantes O, McGuinness MJ, Knaul F. The New World order and international health. BMJ 1997;314:1404-7.
2. Walt G. Globalisation of international health. Lancet 1998;351:434-7.
3. World Health Organization. Cholera in 1997. Wkly Epidemiol Rec 1998;73:201-8.
4. Mhalu FS. Cholera. In: Mwaluko GMP, Kilama WL, Mandara MP, Muru M, Macpherson CNL, editors. Health and disease in Tanzania. London: Harper Collins Academic; 1991. p. 46-55.
5. Health Information and Research Section. Health statistics [abstract]. Tanzania: Ministry of Health; 1997.
6. Benenson AS. Control of communicable diseases in man. 15 ed. Washington: Pan-American Health Organization; 1990. p. 89-94.
7. Barua D, Merson MH. Prevention and control of cholera. In: Barua D, Greennough III WB, editors. Cholera. New York: Plenum; 1992. p. 329-49.

8. Isenberg HD. Clinical microbiology procedures handbook. Washington: American Society for Microbiology; 1992.
9. National Committee for Clinical Laboratory Standards. Performance standards for antimicrobial disk susceptibility tests: approved standard. 4th ed. Villanova (PA): 1990. p. M2-A4.
10. Hughes JM, Boyce JM, Levine RJ, Khan M, Aziz KM, Huq MI, et al. Epidemiology of el Tor cholera in rural Bangladesh: importance of surface water in transmission. Bull World Health Organ 1982;60:395-404.
11. Birmingham ME, Lee LA, Ndayimirije N, Nkurikiye S, Hersh BS, Wells JG, et al. Epidemic cholera in Burundi: patterns of transmission in the Great Rift Valley Lake region. Lancet 1997;349:981-5.
12. Cash RA, Music SI, Libonati JP, Snyder MJJ, Wenzel RP, Hornick RB. Response of man to infection with *Vibrio cholerae*. I. Clinical, serologic, and bacteriologic responses to a known inoculum. J Infect Dis 1974;129:45-52.
13. Levine MM, Black RE, Clemens ML, Nalin DR, Cisneros L, Finkelstein RA. Volunteer studies in development of vaccines against cholera and enterotoxigenic *Escherichia coli*: a review. In: Holme T, Holmegren J, Merson MH, Mollby R, editors. Acute enteric infections in children: new prospects for treatment and prevention. Amsterdam: Elsevier/ North Holland Biomedical Press; 1981. p. 443-59.
14. Webber RH, Mwakalukwa J. The epidemiology of cholera in south-west Tanzania. East Afr Med J 1983;60:848-56.
15. Rodrigues A, Brun H, Sandstrom A. Risk factors for cholera infection in the initial phase of an epidemic in Guinea-Bissau: protection by lime juice. Am J Trop Med Hyg 1997;57:601-4.
